# Associations between paramedics' salutogenic health experience and the intention to stay in the profession: A cross‐sectional survey

**DOI:** 10.1002/nop2.2133

**Published:** 2024-03-18

**Authors:** Eve Hulkkonen, Anne Kouvonen, Hilla Nordquist

**Affiliations:** ^1^ Emergency Medical Services The Wellbeing Services County of Southern Savonia Savonlinna Finland; ^2^ Faculty of Social Sciences University of Helsinki Helsinki Finland; ^3^ Centre for Public Health Queen's University Belfast Belfast Northern Ireland; ^4^ South‐Eastern Finland University of Applied Sciences Kotka Finland

**Keywords:** emergency medical services (EMS), paramedic, personnel turnover, salutogenic health

## Abstract

**Aim:**

To investigate the association between paramedics' salutogenic health and their intentions to stay in their profession.

**Design:**

A cross‐sectional survey.

**Methods:**

Paramedics from eight purposively selected organizations participated in this study (*n* = 433). The data were collected in 2022 with the Salutogenic Health Indicator Scale and validated single questions that assessed work ability, recovery and self‐rated stress. Intention to stay was measured using two questions about the intention to leave prehospital emergency medical service work and leave the nursing sector completely during the next 2 years. Data were analysed using logistic regression and statistical significance tests.

**Results:**

Men had higher mean salutogenic health scores than women. No significant differences in salutogenic health were observed across work experience or occupational groups. Paramedics intending to stay in their profession exhibited higher salutogenic health scores than those intending to leave. In the logistic regression models, increasing levels of salutogenic health and work ability were associated with intention to stay in the profession.

## 1 INTRODUCTION

Emergency medical services (EMS) manage a range of rapid and unpredictable situations and conditions (Lawn et al., 2020). EMS work is psychosocially demanding (Ericsson et al., 2022), as paramedics are routinely exposed to suffering, pain and death. In addition to lifesaving interventions, paramedics' work extends to handling non‐acute patient situations, social issues and other emergencies (Andrew et al., 2020; Magnusson et al., 2020; Pekanoja et al., 2018), leading to potential psychological distress (Ericsson et al., 2021). Moreover, EMS typically involves long shifts operating around the clock. These irregular working hours can disrupt the natural sleep–wake rhythms and are linked to health issues, such as cardiovascular diseases, anxiety, fatigue and sleep disorders (Khan et al., 2021; Nowak & Lukomska, 2021).

The physical health and health‐related lifestyle factors of paramedics, including physical activity and nutrition, are crucial to their overall well‐being (Rice et al., 2014). While work can be physically demanding, approximately half of all shifts are somewhat sedentary. Such physical demands increase the risk of occupational injuries and chronic pain among paramedics. Concurrently, long shifts and the hectic nature of work, coupled with the resulting fatigue, can hinder healthy lifestyles (Ericsson et al., 2022; Rice et al., 2014).

The high levels of depression, anxiety and stress, experienced by paramedics escalated during the COVID‐19 pandemic (Huang et al., 2022). The support provided by EMS organizations has been deemed insufficient (Ericsson et al., 2022; Lawn et al., 2020). Evidence suggests that the pandemic has increased the intention of frontline healthcare workers, including paramedics, to leave (De Los Santos & Labrague, 2020; Labrague, & de los Santos, J., 2021; Mousavi et al., 2022). For example, in a US survey of 9688 employees from 258 EMS organizations, 20%–30% of paramedics and emergency medical technicians expressed an intention to leave the profession. A turnover rate of 25% implies a complete replacement of an organization's EMS staff every 4 years (Lawrence, 2021). Additionally, nurses and paramedics have lamented poor communication with employers, a lack of respect for their work, and inadequate personal security measures during the pandemic (Christianson et al., 2023; Thielmann et al., 2022). Furthermore, dissatisfaction with salaries had increased during the pandemic (Skrikanht et al., 2022). Collectively, these factors contribute significantly to paramedics' increasing desire to leave their profession (Skrikanht et al., 2022).

The health of healthcare professionals has been predominantly studied from the perspective of ill health. Existing research on paramedics' health primarily focuses on the negative aspects, associating their poor overall health perception, physical condition and particularly high levels of perceived stress with their intention to leave their profession (see Blau & Chapman, 2016; Mirzaei et al., 2021; Skrikanht et al., 2022). Nevertheless, emerging evidence suggests that a good quality of work life, employee participation in decision‐making (Aminizadeh et al., 2022) and strong organizational support, such as managerial endorsement of a healthy work–life balance (Ma et al., 2022; Petrie et al., 2018; Sanner‐Stiehr et al., 2022), can positively influence the intention to remain in demanding nursing roles. Likewise, recent systematic reviews have identified that overall job satisfaction (de Vries et al., 2023; Pressley & Garside, 2023), favourable career development opportunities and a sense of work–life balance (de Vries et al., 2023) enhance retention rates in the nursing sector. This positive perspective requires further exploration, especially in the context of paramedics facing challenging working conditions (Ericsson et al., 2022; Lawn et al., 2020; Rice et al., 2014). There is also a recognized need for empirical studies that adopt a more holistic health perspective and consider individual experiences (Bringsén et al., 2009; Hult & Välimäki, 2023).

The concept of salutogenesis shifts the focus away from understanding health as an absence of illness. Salutogenesis emphasizes coping resources at both individual and community levels, centred on personal health experiences (Bringsén et al., 2009). A key element related to salutogenesis is the sense of coherence, a life attitude that assists individuals in better coping with adversities and stress. A strong sense of coherence enables individuals to perceive their lives as comprehensible, manageable and meaningful, fostering their belief that life will proceed well (Mittelmark, 2022). Additionally, the salutogenic approach beneficially guides practical health promotion by focusing on enhancing capabilities rather than addressing deficiencies (García‐Moya & Morgan, 2017; Morgan & Hernán, 2013).

A Swedish research team developed The Salutogenic Health Indicator Scale (SHIS), which embodies a salutogenic and holistic health perspective based on theories of health and well‐being. In the SHIS, health is perceived as a sense of well‐being within oneself, which is assessable through physical, mental and social well‐being experiences and feelings. Positive experiences and emotions are seen as beneficial to health, with health itself aiding in navigating life challenges and pursuing personal goals. The impact of an illness is considered in terms of its potential to restrict an individual's functional capacity (Bringsén et al., 2009).

The SHIS has limited applications in healthcare research. Recently, the SHIS has been utilized in a longitudinal study of Finnish care workers (Hult & Välimäki, 2023), Swedish primary healthcare employees (Ejlertsson et al., 2018), Swedish municipal elderly care employees (Persson et al., 2018), Swedish public dental health employees (Lindmark et al., 2018), and Lithuanian hospital employees (Andruškienė et al., 2015). However, research exploring paramedics' salutogenic health experiences remains scarce, particularly studies linking these experiences to their intentions to stay in the profession. Therefore, this study investigated the association between paramedics' salutogenic health and their intention to stay in their profession. Our research questions were as follows: (1) Are there differences in salutogenic health by gender, work experience or occupational group? (2) How is salutogenic health associated with intention to stay in the profession? The results of this study can be used to improve strategies to foster paramedics' overall well‐being and to retain experienced staff in their profession.

## 2 MATERIALS AND METHODS

### 2.1 Design

This was a survey‐based cross‐sectional study targeting the Finnish basic‐ and advanced‐level paramedics and EMS field supervisors.

### 2.2 Setting

Finland has two operational levels of paramedics: basic and advanced. Basic‐level paramedics are practical nurses with prehospital specialization, or Registered Nurses without prehospital specialization. Advanced‐level paramedics are Registered Nurses with a 30 ECTS (European Credit Transfer System), advanced‐level prehospital specialization or an emergency care bachelor's degree. Working in the EMS and nursing sectors requires registration with the Finnish National Supervisory Authority for Welfare and Health (Valvira) (Dúason et al., 2021). EMS field supervisors are experienced, and advanced‐level paramedics are operational leaders in the EMS. They ensure the functional use of ambulances in the field and, for example, lead to multi‐patient situations and, when necessary, participate in high‐acuity situations. The work of paramedics is demanding, as they evaluate patients' care needs and provide treatment independently based on existing instructions and protocols. The work of basic‐level paramedics includes assessing the need for treatment, administering simple medication(s) and arranging transportation. Advanced‐level paramedics can, for example, perform electrical cardioversion and initiate intravenous medication. For guidance in decision‐making or complex medical treatments, paramedics can consult physicians who may also join them in the field during critical situations.

### 2.3 Sampling

The inclusion criteria (target group) for the study were: (1) basic‐ or advanced‐level paramedics or EMS field supervisors; and (2) working in prehospital EMS in an organization that granted research permission for the study.

Research permits were requested and received from eight Finnish EMS organizations from four (out of five) different special responsibility areas. The organizations in question were selected based on size (more than 80 employees) and location (from different parts of Finland, larger and smaller cities, and rural areas).

### 2.4 Instruments

#### 2.4.1 Background questions

Gender (woman, man, other), age (in years) and work experience (in years) were included as background questions. In addition, four responses were provided for the professional group to accommodate the diverse range of professional titles in the EMS field: ‘paramedic, basic‐level’, ‘paramedic, advanced‐level’, ‘EMS field supervisor’ and ‘other, please clarify’.

#### 2.4.2 Salutogenic health indicator scale

The data were collected using the SHIS‐FI (the Finnish version, the permission for usage being granted by the developer Dr. Å. Bringsén), designed to measure well‐being at work from a positive perspective (Bringsén et al., 2009; Hult & Välimäki, 2023). The SHIS was initially validated among the hospital staff (*n* = 483) with two identified factors: intrapersonal characteristics (Cronbach's alpha (*α*) = 0.90) and interactive functions (*α* = 0.84). The overall internal consistency was excellent (*α* = 0.92) (Bringsén et al., 2009). Further validation using cognitive interviews (*n* = 14) confirmed its high qualitative validity (Nilsson Lindström et al., 2018). The SHIS‐FI has recently been validated in Finnish care workers (*n* = 2117), showing excellent internal consistency (*α* = 0.94) (Hult & Välimäki, 2023).

The SHIS‐FI has 12 questions rated on a scale of 6–1 (6 indicating a positive response and 1 indicating a negative response). The possible score range is 12–72, with higher scores indicating better salutogenic health (Bringsén et al., 2009; Hult & Välimäki, 2023). In this study, standardized SHIS scores of 0–100 were used, where a score of ≤55.0 indicated a poorer perception of health, implying that health promotion interventions are needed. A score of 55.1–70.0 indicates that there is potential to improve the perception of health, and that health promotion interventions are further needed. The score of 70.1–80.0 indicated a relatively positive perception of health in the group, with room for health improvement. A score of >80.0 indicated a positive perception of health (Bringsén, 2022).

#### 2.4.3 Intention to leave the profession

Two statements addressed the intention to leave the profession: ‘I am seriously considering leaving prehospital EMS work during the next 2 years (moving to other positions in the nursing sector)’ and ‘I am seriously considering leaving the nursing sector completely during the next 2 years’. The response options were ‘Yes’, ‘No’ and ‘Not sure’.

#### 2.4.4 Recovery, stress and work ability

An individual's self‐evaluation of recovery (‘How well do you generally feel you're able to recover from the strain caused by your job after the working day?’) was measured utilizing a 5‐point Likert scale ranging from 1 (very well) to 5 (very poorly) (Kinnunen et al., 2011). Self‐rated stress (‘Stress is a situation in which a person feels tense, restless, nervous, or anxious or cannot sleep at night because their mind is troubled all the time. Do you currently feel this kind of stress?’) on a 5‐point Likert scale ranging from 1 (not at all) to 5 (very much) (Elo et al., 2003). The single‐item question ‘current work ability compared with your personal best’ was measured with a possible score of 0 (‘completely unable to work’) to 10 (‘work ability at its best’) (Ahlstrom et al., 2010).

### 2.5 Data collection

Invitations to participate in the study, a link to the research privacy notice and a link to the Webropol questionnaire were sent to the target group between April and September 2022 through contact persons either in a supervisory position or working in HR designated by the organizations. Based on the information received from contact persons, the invitation links reached 1189 target group members. The contacts sent two reminders once per week. The data collection ended in October 2022. In all, 437 paramedics and EMS field supervisors answered the survey (response rate: 37%). Incomplete responses were excluded (*n* = 4). The final analysis included data from 433 participants.

### 2.6 Ethical considerations

Participation in the study was voluntary, and informed consent was obtained. The participants' identities were protected by limiting the number of background information questions; therefore, identifying individuals was not possible. As stated in the research permit applications and research privacy notice for participating paramedics, the participating organizations were also not reported.

### 2.7 Statistical methods

Background characteristics were reported as percentages. Age was categorized into six groups, and work experience was categorized into five. In the background variable of the professional group, among the ‘other’ respondents (*n* = 25), firefighter–paramedics were included in the same category as basic‐level paramedics, unless the participant specified their current duties were classified as advanced level. Participants who clarified that they worked in various supervisory or management positions were included in the same category as EMS field supervisors.

Based on the extent of experience, descriptive results for recovery, self‐rated stress and work ability were analysed in two groups: <10 years of work experience and ≥10 years of work experience. The mean, standard deviation (SD), median and interquartile range (IQR) are reported. Statistical differences were tested using the Mann–Whitney *U* test, with a significance level of *p* < 0.05.

The two questions regarding the intention to leave the profession were analysed from a positive angle, that is, the intention to continue their profession. First, the intention to stay in the profession was analysed based on work experience and presented for both questions and all response options. In addition, the summary results were presented for those who answered ‘Yes’ (i.e. intention to leave) to both questions and ‘No’ (i.e. intention to stay) respectively. Statistical differences were tested using Pearson's chi‐square test with a significance level of *p* < 0.05.

In this study, the SHIS‐FI had excellent internal consistency (*α* = 0.93). The answers were summed following the analysis instructions, and the sum value was standardized in accordance with Bringsén (2022) by calculating 100 × (sum value‐12)/(72–12). The salutogenic health scores by gender (women/men), two work experience groups and professional groups (only basic‐level/advanced‐level paramedics) were presented as means and SD. Salutogenic health by intention to stay in or leave the profession (‘not sure’ answers were excluded) was reported with mean and SD. Statistical differences and their 95% confidence intervals (CIs) were tested using an independent sample t‐test with a significance level set at *p* < 0.05.

The associations between the independent variables and ‘yes’ intention to leave prehospital EMS work and ‘yes’ intention to leave the nursing sector completely were explored using a logistic regression model. The group of potential predictors (professional group, gender, work experience, salutogenic health score, recovery, self‐rated stress and work ability) was first narrowed down using stepwise logistic regression. The only significant predictors were salutogenic health and work abilities. The results are reported in four models adjusted for length of work experience and gender. Odd ratios with their 95% CIs are reported.

All the analyses were performed with SPSS Inc., Chicago, IL, USA, version 28.

## 3 RESULTS

### 3.1 Background information

More men (57%, *n* = 247) than women (41%, *n* = 177) participated in this study (Table 1). Most participants were 30–34 years old (24%, *n* = 107), and advanced‐level paramedics was the largest occupational group (68%, *n* = 293). The participants included those who were new to the profession and experienced professionals. One‐fourth (*n* = 109) of the participants had <5 years of work experience, whereas 13% (*n* = 56) had >20 years of experience.

**TABLE 1:** Participant background information, *n* = 433.

	%	*n*
Gender		
Women	40.9	177
Men	57.0	247
Other or missing	2.1	9
Age, years		
<25	4.8	21
25–29	19.4	84
30–34	24.7	107
35–39	19.2	83
40–44	15.2	66
≥45	16.6	72
Professional group		
Paramedic or firefighter–paramedic, basic level	26.6	115
Paramedic or firefighter–paramedic, advanced level	67.7	293
EMS field supervisor or other EMS manager	5.8	25
Work experience in EMS, years		
<5	25.2	109
5–9	25.4	110
10–14	23.3	101
15–19	13.2	57
≥20	12.9	56

Paramedics with <10 years of work experience reported better work ability than those who had worked for ≥10 years (mean 8.56 vs. 7.86, *p* = <0.001; Table 2). Similarly, paramedics with less work experience reported better recovery after a shift [mean 2.22 versus 2.52] (lower means being better, *p* = 0.006). Self‐rated stress was similar between the two work experience groups (mean 2.5 vs. 2.43, *p* = 0.387).

**TABLE 2:** Recovery, self‐rated stress and work ability among Finnish paramedics by work experience.

	<10 years of work experience, *n* = 219		≥10 years of work experience, *n* = 214		*p* ^b^
	Mean (SD)	Md (IQR)	Mean (SD)	Md (IQR)	
Work ability, *n* = 431^a^ (0 = completely unable to work; 10 = work ability at its best)	8.56 (1.47)	9 (8.10)	7.86 (1.87)	8 (7.9)	<0.001
Recovery, *n* = 433 (1 = very well; 5 = very poorly)	2.22 (0.89)	2 (2.3)	2.52 (1.06)	2 (2.3)	0.006
Stress, *n* = 433 (1 = not at all to 5 = very much)	2.5 (0.90)	2 (2.3)	2.43 (0.91)	2 (2.3)	0.387

^a^ Missing=2.

^b^ Statistical significance of the difference between work experience groups, Mann–Whitney *U*‐test.

More participants intended to stay in prehospital EMS work than in the nursing sector (Table 3). Paramedics with less work experience were more likely to stay in prehospital EMS work (72%) than those with more work experience (61%) (*p* = 0.037).

**TABLE 3:** Intention to stay in the nursing sector or prehospital EMS work among Finnish paramedics by work experience, %.

Intention to stay in	<10 years of work experience, *n* = 219%	≥10 years of work experience, *n* = 214%	*p* ^a^
Nursing sector			
Yes	53.0	47.2	0.451
No	32.0	34.6	
Not sure	15.0	18.2	
Prehospital EMS work			
Yes	72.1	61.2	0.037
No	16.4	25.7	
Not sure	11.4	13.1	
Summary			
Yes, to both	47.9	42.1	0.126
No, to both	11.9	16.4	

^a^ Statistical significance, Pearson Chi‐square test.

### 3.2 Salutogenic health and the intention to stay in the profession

Men had higher mean salutogenic health scores than women (68.88 versus 62.41, *p* < 0.001; Table 4). The mean salutogenic health score was marginally higher among those with <10 years of work experience (67.72) than among those with ≥10 years of work experience (64.60); however, the difference was not statistically significant. The mean salutogenic health score was higher among basic‐level paramedics (68.35) than advanced‐level paramedics (64.65), but the difference was not statistically significant.

**TABLE 4:** Salutogenic health of Finnish paramedics (index value 0–100) by gender, work experience and professional group.

	Mean (SD)	*p* ^a^	95% CI^b^	
			Lower	Upper
Gender				
Women *n* = 177	62.41 (17.85)	<0.001	−9.77	−3.17
Men *n* = 247	68.88 (16.48)			
Work experience				
<10 years, *n* = 219	67.72 (16.23)	0.062	−0.16	6.41
≥10 years, *n* = 214	64.60 (18.49)			
Professional group				
Paramedic or firefighter–paramedic, basic level, *n* = 115	68.35 (16.02)	0.056	−0.10	7.50
Paramedic or firefighter–paramedic, advanced level, *n* = 293	64.65 (18.12)			

^a^ Statistical significance, Independent samples *t*‐test.

^b^ 95% Confidence interval of the mean difference.

Table 5 shows that the paramedics who intend to stay in the nursing sector or prehospital EMS work had higher salutogenic health scores than paramedics who intended to leave (mean 71.28 vs. 59.21 and mean 70.40 vs. 55.09, respectively, *p* ≤ 0.001).

**TABLE 5:** Salutogenic health of Finnish paramedics (index value 0–100) and intention to stay or leave the nursing sector, prehospital EMS work, or both.

	Intention to stay Mean (SD)	Intention to leave Mean (SD)	*p* ^a^	95% CI^b^	
Nursing sector	*n* = 217	*n* = 144		Lower	Upper
	71.28 (15.20)	59.21 (18.73)	<0.001	−15.56	−8.58
Prehospital EMS work	*n* = 289	*n* = 91			
	70.40 (15.26)	55.09 (18.41)	<0.001	−19.10	−11.51
Both	*n* = 195	*n* = 61			
	72.74 (13.81)	52.90 (17.91)	<0.001	−24.15	−15.55

^a^ Statistical significance, Independent samples *t*‐test.

^b^ 95% Confidence interval of the mean difference.

Table 6 presents the results of the logistic regression analyses. Model 2, adjusted for work experience and gender, showed that when the salutogenic health score increased by one point, the odds of intention to leave the nursing sector were 2% lower (OR 0.98, 95% CI 0.96, 0.99). When work ability increased by one point, the odds of an intention to leave the nursing sector were 19% lower (OR 0.81, 95% CI 0.70, 0.95). Similarly, salutogenic health and work ability were associated with a decreasing intention to leave prehospital EMS work (OR 0.97, 95% CI 0.95, 0.99 and OR 0.77, 95% CI 0.66, 0.91, respectively, in model 2).

**TABLE 6:** Logistic regression analysis: statistically significant predictors of intention to leave the nursing sector completely and intention to leave prehospital EMS work, adjusted for gender and work experience (OR = Odds Ratio, CI = 95% Confidence Intervals, *p* = statistical significance).

	Model 1^a^				Model 2^a^			
	OR	Lower CI	Upper CI	*p*	OR	Lower CI	Upper CI	*p*
Nursing sector								
Salutogenic health	0.97	0.96	0.98	<0.001	0.98	0.96	0.99	0.004
Work ability					0.81	0.70	0.95	0.009
Prehospital EMS work								
Salutogenic health	0.96	0.94	0.97	<0.001	0.97	0.95	0.99	<0.001
Work ability					0.77	0.66	0.91	0.002

^a^ Adjusted for gender and work experience.

## 4 DISCUSSION

This study investigated the association between Finnish paramedics' salutogenic health and their intentions to stay in their profession. As per the main findings, men had higher mean salutogenic health scores than women. No significant differences in salutogenic health were observed across work experience or occupational groups. Salutogenic health and work ability were associated with intention to stay in the profession. When salutogenic health increased, the intention to leave decreased, and when work ability increased, the intention to leave decreased.

In this study, the overall salutogenic health experience was higher among paramedics than in a previous study of Finnish care workers (Hult & Välimäki, 2023). However, an analysis of demographic factors revealed that paramedics have considerable opportunities to improve their perceptions of health. Experiencing high levels of salutogenic health is a key characteristic of professionally thriving nurses (Stock, 2017). This concept reflects a strong sense of coherence and perceives life as comprehensible, manageable and meaningful (Hult & Välimäki, 2023).

Generally, the Finnish paramedics are highly educated, feel empowered and successfully apply their extensive training and special competencies (Ericsson et al., 2022). Positive mental well‐being is often supported by strong resilience and a significant sense of workplace belonging. Peer support, communication and social support within the organization are crucial for paramedics' overall well‐being and are commonly found in EMS work (Abbaspour et al., 2020; Ericsson et al., 2022; Petrie et al., 2018). Additionally, quality of work life is linked to organizational commitment (Aminizadeh et al., 2022). Paramedic work involves various factors that may negatively affect health and well‐being. These include shift work, long hours, constant readiness for stressful events, physically demanding tasks such as lifting and carrying patients in challenging conditions, and working on the frontlines during situations such as the COVID‐19 pandemic. These conditions can lead to fatigue, post‐traumatic stress, depression and musculoskeletal injuries (Angehrn et al., 2020; Blanchard et al., 2022; Ericsson et al., 2021, 2022).

The results of our study indicate that work ability and recovery may be better among less experienced paramedics; however, further research is needed to confirm this. Nonetheless, as paramedics age and gain work experience, the challenges of prehospital EMS work can become more burdensome (Eiche et al., 2019; Nowak & Lukomska, 2021). Most paramedics are relatively young (Abbaspour et al., 2020; Taylor et al., 2022), and Nowak and Lukomska (2021) suggested that the chronic negative effects of demanding EMS work may become significantly harmful to paramedics' health, particularly as they reach middle age (Eiche et al., 2019; Nowak & Lukomska, 2021). Salutogenic health experiences highlight a person's mobilizable resources (Hult & Välimäki, 2023), which, in this context, could reflect the ability to adapt to different stressors. Our results revealed that promoting the health of paramedics with several years of work experience is important, as their intention to leave seems to increase with work experience. Although current and previous studies have shown that younger paramedics have better work‐related health (Eiche et al., 2019), attention must also be paid to their health and overall well‐being at the beginning of their careers to manage demanding work and commitment to their profession.

The high turnover rate of paramedics is a global challenge for EMS organizations (Blau & Chapman, 2016; Dopelt et al., 2019), and the situation further deteriorated during the COVID‐19 pandemic (Mirzaei et al., 2021). This poses a significant risk to patient safety and quality of care (Blau & Chapman, 2016; Dopelt et al., 2019). Previous studies have shown that stress and low job satisfaction have a strong negative impact on paramedics' intention to stay (Blau et al., 2016). Our findings are in line with those of previous studies that reported the intention of paramedics to stay in their profession, showing a higher experience of salutogenic health exceeding the threshold for a relatively positive perception of health, as opposed to paramedics with the intention to leave. Our results suggest that promoting paramedics' positive health and well‐being and focusing on boosting their capabilities could be key elements in retaining paramedics in their profession. All contributions to the well‐being and health of personnel are significant, as they are among the most important elements affecting an organization's productivity. When losing experienced staff, an organization loses the skills and knowledge of experienced paramedics, and the solution is to recruit new, inexperienced paramedics, which causes extra work along with recruitment and training costs (Blau & Chapman, 2016; Lawrence, 2021). Paramedic education is usually long; for instance, advanced‐level training in Finland lasts for 4 years at the University of Applied Sciences (Dúason et al., 2021). Therefore, newly graduated paramedics are not immediately available to address the shortage of professionals in EMS organizations.

Finnish paramedics, beyond their prehospital EMS roles, qualify for various positions in the nursing sector. Our study showed that more participants intended to stay in prehospital EMS work than in the nursing sector. This preference may partly be due to a unique ‘paramedic role identity’ that encompasses caregiving, thrill‐seeking, capacity, and duty (Donnelly et al., 2015). Paramedic education, particularly a broad bachelor's degree, reinforces this identity by focusing on urgent care situations (Ericsson et al., 2022). This role identity differs significantly from that of nurses in other fields (Donnelly et al., 2015; Mausz et al., 2022). Specialized professionals capable of demanding work have options to address the current shortage of nursing personnel. For instance, Ma et al. (2022) found that many emergency care nurses have the intention to leave, and in their article, the authors also discussed the opportunities that professional expertise offers. It can be argued that intensive care nurses are in a position similar to that of highly educated Finnish paramedics, implying that they can choose where to work.

However, the nature of prehospital EMS work has changed from dealing solely with life‐threatening emergencies to helping older people with non‐urgent situations and social emergencies (Andrew et al., 2020; Magnusson et al., 2020; Pekanoja et al., 2018). Thus, for many paramedics, there could be a discrepancy between their assumed and actual role identities, which could cause feelings of not fitting in. It is possible that, to some extent, the reality of the paramedic profession can be disappointing and lead to dissatisfaction and an intention to leave. Despite this, pre‐hospital EMS work may still be perceived as more appealing than other nursing jobs. The intention to leave may stem more from a high overall workload and resource scarcity (Blau & Chapman, 2016; Ericsson et al., 2022; Mirzaei et al., 2021) than job dissatisfaction. Given these changes, it seems prudent to adapt paramedic education to better equip paramedics to maintain personal health and workplace well‐being and to align paramedic identity with the evolving nature of the profession (Carnicelli et al., 2023; Mausz et al., 2022).

### 4.1 Strengths and limitations

Although the study had several strengths, such as the fact that the participants were mostly experienced paramedics from various EMS organizations in different areas of Finland, its limitations must be considered. For instance, the response rate was 37%, and thus not much precise information was available for the entire population. However, calculating with the corrected sample size formula for finite populations, 291 responses were required to achieve a 95% CI with a 0.05 margin of error. Thus, 433 responses were sufficient. Furthermore, the cross‐sectional study design had limitations, and causal conclusions could not be drawn.

The strength of this study lies in the use of validated instruments (Ahlstrom et al., 2010; Bringsén et al., 2009; Elo et al., 2003; Hult & Välimäki, 2023; Kinnunen et al., 2011; Nilsson Lindström et al., 2018). Although the strength of the SHIS is its short length, which improves its usability, it still considers nine different dimensions of health and provides a holistic impression of salutogenic health. However, there is a risk that a limited scale may miss some important dimensions (Bringsén et al., 2009), although previous studies (Ejlertsson et al., 2018; Hult & Välimäki, 2023; Lindmark et al., 2018) have indicated that the SHIS is a useful instrument for measuring salutogenic health. In this study, the SHIS‐FI showed excellent internal consistency.

This study focused only on the association between salutogenic health experiences and the intention to stay, and there could be many other factors behind the intention to stay or leave the profession. In the logistic regression analysis, Nagelkerke's R2 values were low (to 0.135–0.217), suggesting that the model explained only a small portion of the variance in the dependent variable. Therefore, future studies should incorporate a broader range of relevant variables to improve the overall fit of the model. According to previous findings, quality of work life, employee participation in decision‐making (Aminizadeh et al., 2022) and different forms of organizational support (Ma et al., 2022; Petrie et al., 2018; Sanner‐Stiehr et al., 2022) are potential explanatory variables. Nonetheless, logistic regression analysis indicated that salutogenic health and workability were explanatory variables to consider, thus providing a foundation for further research. Considering these factors, the results of this study can be regarded as indicative of and supporting future research. However, the results of this study emphasize the importance of the overall health experience, thereby adding to the knowledge of a phenomenon that has been studied to a limited extent among paramedics. These findings encourage further research on salutogenic health in the field of EMS.

### 4.2 Conclusion

Better salutogenic health experiences were associated with the intention to stay in the paramedic profession. The results of this study underscore the necessity for strategies that promote paramedics' health and well‐being to improve retention rates and ensure high‐quality patient care. Furthermore, there is a need for studies focusing on positive health factors among nurses in general rather than just ill health and examining nurses' intentions to stay in the nursing sector. Utilizing the salutogenic approach would also offer deeper insights into the practical utility of health promotion activities in this professional group.

## AUTHOR CONTRIBUTIONS

EH: Formal Analysis, Investigation, Visualization, Writing—original draft. AK: Methodology, Resources, Supervision, Writing—review and editing. HN: Conceptualization, Data curation, Formal Analysis, Investigation, Methodology, Project administration, Resources, Supervision, Visualization, Writing—review and editing.

## FUNDING INFORMATION

This research received no specific grants from any funding agency in the public, commercial or not‐for‐profit sectors.

## CONFLICT OF INTEREST STATEMENT

The authors declare that they have no competing interests.

## ETHICAL APPROVAL

The Ethics Committee of the South‐Eastern Finland University of Applied Sciences evaluated the research plan and provided a favourable ethical statement for the study on March 28, 2022. The Ethics Committee did not provide specific numbers for their statements.

## Data Availability

Research data are not shared.
